# COVID-19 Pandemic in Lebanon: One Year Later, What Have We Learnt?

**DOI:** 10.1128/mSystems.00351-21

**Published:** 2021-04-20

**Authors:** Jad Koweyes, Tamara Salloum, Siwar Haidar, Georgi Merhi, Sima Tokajian

**Affiliations:** a Department of Natural Sciences, School of Arts and Sciences, Lebanese American University, Byblos, Lebanon; Istanbul Medipol University School of Medicine

**Keywords:** COVID-19, epidemiology, Lebanon, pandemic, public health, SARS-CoV-2

## Abstract

Lebanon is witnessing an unprecedented crisis with the rapid spread of coronavirus disease 2019 (COVID-19), financial meltdown, economic collapse, and the Beirut Port explosion. The first wave began in February 2020, following which the country experienced several episodes and peaks while alternating between lockdowns and phased liftings. One year of the pandemic revealed that effective mitigation could not be separated from the collapse of the ongoing economic, political, and health sectors. Scaling up vaccination, preparedness, and response capacities is essential to control community transmission. The World Health Organization (WHO), National Council for Scientific Research—Lebanon (CNRS-L), nongovernmental organizations (NGOs), and humanitarian responses proved to be the safety net for the country during the current pandemic.

## PERSPECTIVE

## COVID-19

On 21 December 2019, hospitals in Wuhan, China, witnessed an increased number of patients suffering from flu-like symptoms (1). Within a few days, on 27 December 2019, a Chinese lab assembled an almost complete genome of the virus and named it 2019-nCoV; this virus showed 96.2% sequence similarity to a bat severe acute respiratory syndrome-related coronavirus (SARS-CoV; RaTG13) collected in Yunnan province in China (2) and 79% and 50% similarities to SARS-CoV and Middle East respiratory syndrome MERS-CoV, respectively. The authorities initially kept the information classified until 30 December 2019, when warnings began on social media. On 9 January 2020, a novel coronavirus was linked to the outbreak, which coincided with an important catalyst for the rapid viral spread, the “chunyun,” the world’s largest human migration which happens before the lunar new year, with approximately 3.6 billion trips from, to, and within China (3). On 13 January 2020, the first case outside China was detected in Thailand (4). By the end of the month, the virus had already reached 17 countries, following which WHO declared COVID-19 as a public health emergency of global concern and worldwide pandemic by 11 March 2020 (5).

## COVID-19 AND LEBANON

**FIG 1:**
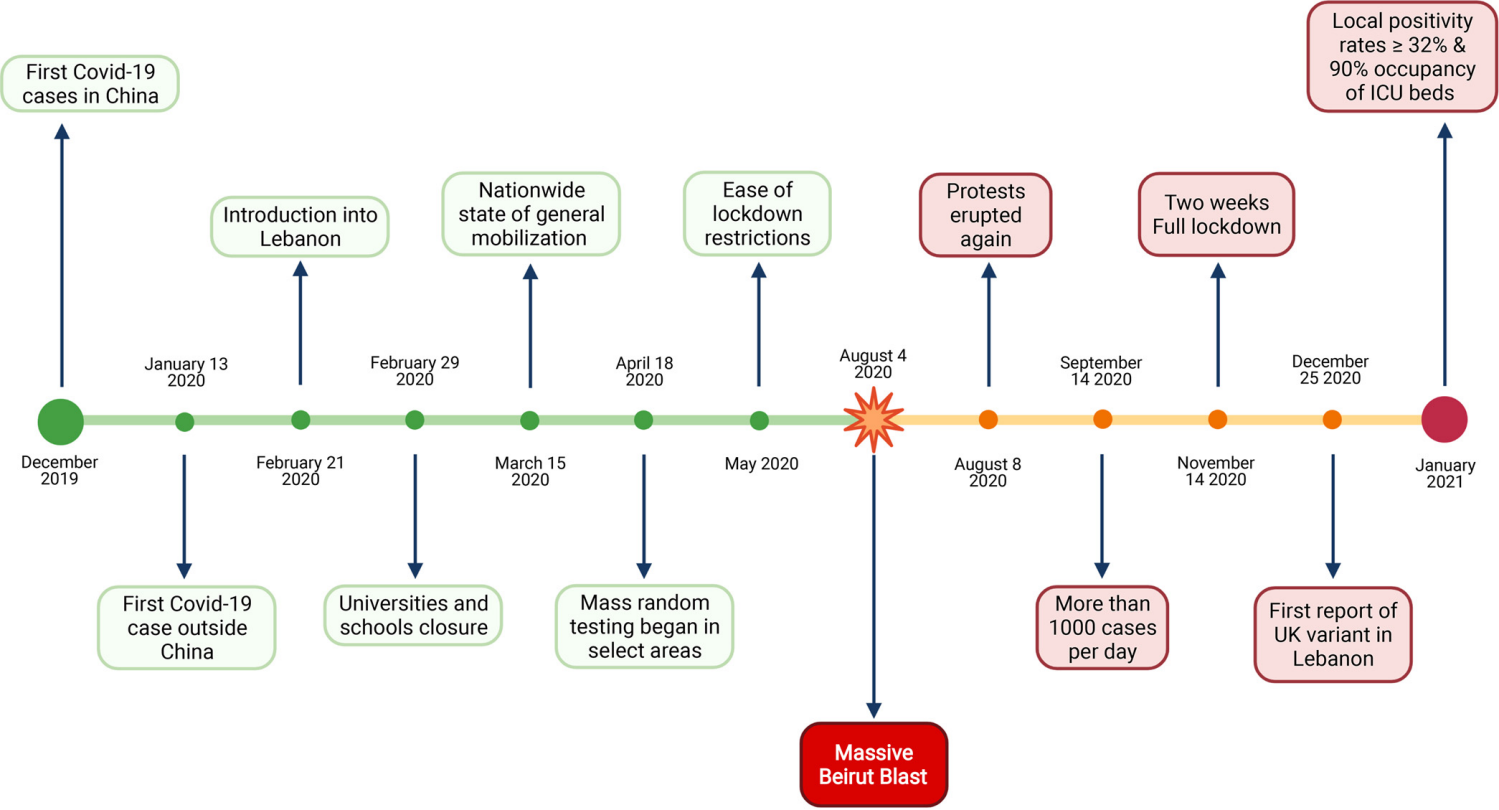
COVID-19 timeline in Lebanon and major associated events. Created using BioRender.

**FIG 2:**
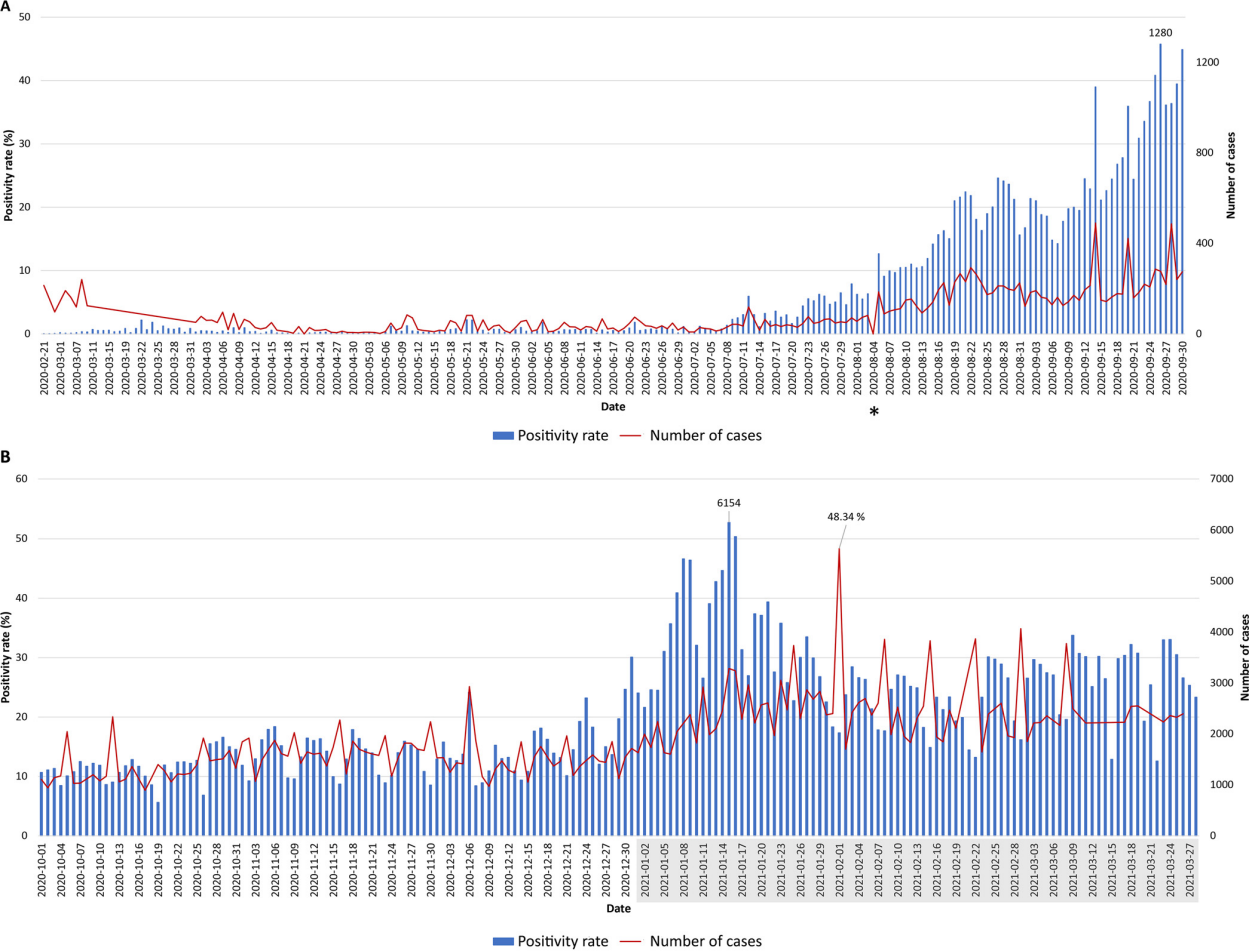
The number of new daily COVID-19 cases and the daily positivity rate over a 1-year period. (A) Data shown covered the period between 21 February 2020 to 30 September 2020; (B) data shown covered the period between 10 January 2020 to 27 March 2021 (11) (https://www.emro.who.int/countries/lbn/index.html and https://twitter.com/WHOLebanon). Only dates with reported COVID-19 cases were included. The Beirut Port explosion on 4 August 2020 is indicated by an asterisk. 2021 dates are shown shaded in gray.

The first COVID-19-positive case was confirmed in Lebanon on 21 February 2020; the first case was a 45-year-old woman from Iran, while only suspected cases from the same flight were tested (6). Four days later, travel restrictions were imposed, reducing flights from high-risk areas, and lockdown measures were taken starting with the closure of all universities and schools (7). A nationwide strict lockdown plan was set following the first severe acute respiratory syndrome coronavirus 2 (SARS-CoV-2)-related death on 10 March 2020 and which was associated with the WHO’s announcement that the country entered stage three of the transmission. The time lapse between the set-up and plan implementation was associated with a dramatic increase in the number of people testing positive for the virus (Fig. 1 and 2).

## FIRST LOCKDOWN

During the first phase of the lockdown, the numbers of test centers and PCR tests performed were limited. The positivity rate reported during this period was correlated with the number of tests performed which in general was very limited. The average reported daily cases was 16 in the period extending from 15 March to 17 April 2020 (Fig. 2A). Mass random testing was initiated in April within selected targeted areas. The number of detected COVID-19-positive tests was low, culminating in easing of the lockdown measures the first week of May (Fig. 1).

## EASING LOCKDOWN AND BEIRUT PORT EXPLOSION

Measures were eased, and the Beirut International airport was reopened on 1 July 2020. Relaxing the measures led to a surge in the daily number of reported cases, with the positivity rate remaining below 5% (Fig. 2A). On 4 August 2020, a massive blast shook the heart of Beirut and was ranked the third-largest explosion of modern time, killing more than 220, injuring 6,500, and displacing 300,000 people (8). Major hospitals in Beirut, Lebanon, were severely damaged. The COVID-19 hospital wards were demolished. The remaining functional hospitals were overwhelmed treating patients injured in the blast; the country started facing the third COVID-19 wave (Fig. 2B). As of 14 September 2020, the numbers were still increasing, surpassing 1,000 cases/day (the equivalent of 159.4 cases/million), with the positivity rate reaching as high as 13.9% during the first 2 weeks of November. An immediate lockdown was imposed and extended until 30 November 2020 with no significant changes in the volume of testing and positivity rates (Fig. 2B).

## UK VARIANT

**FIG 3:**
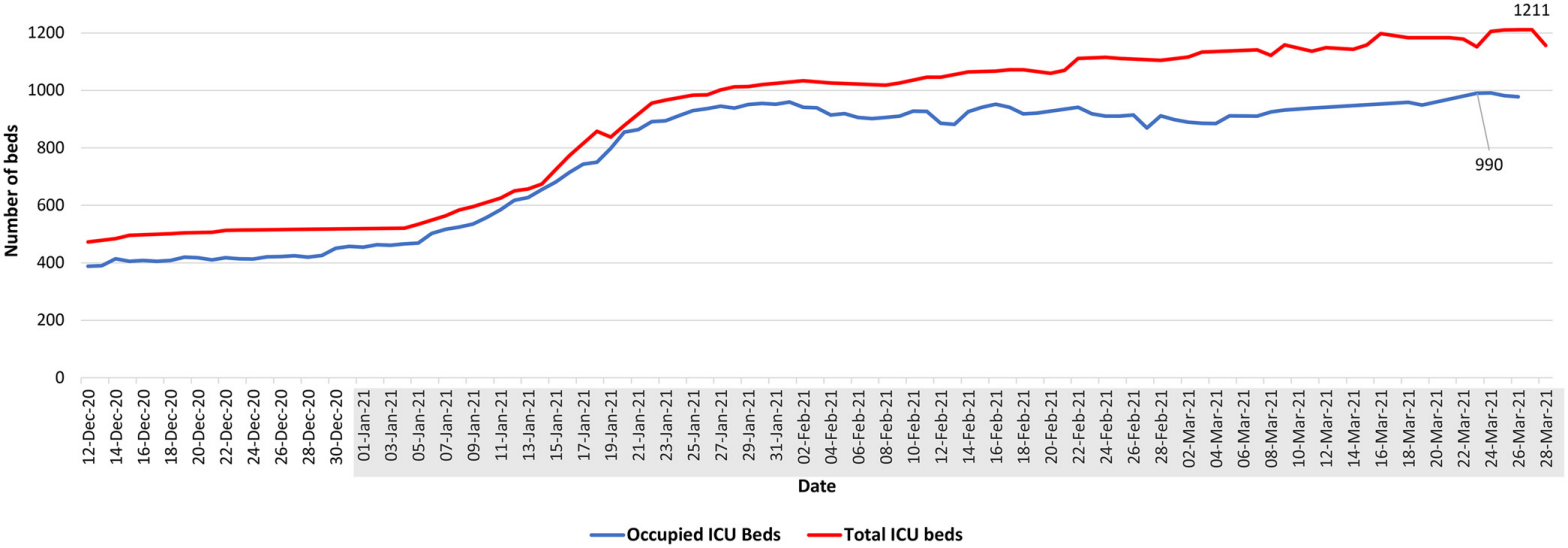
Daily occupancy of hospital ICU beds and total ICU bed capacities. Data shown covered the period between 12 December 2020 to 28 March 2021 (https://www.emro.who.int/countries/lbn/index.html and https://twitter.com/WHOLebanon). 2021 dates are shown shaded in gray.

**FIG 4:**
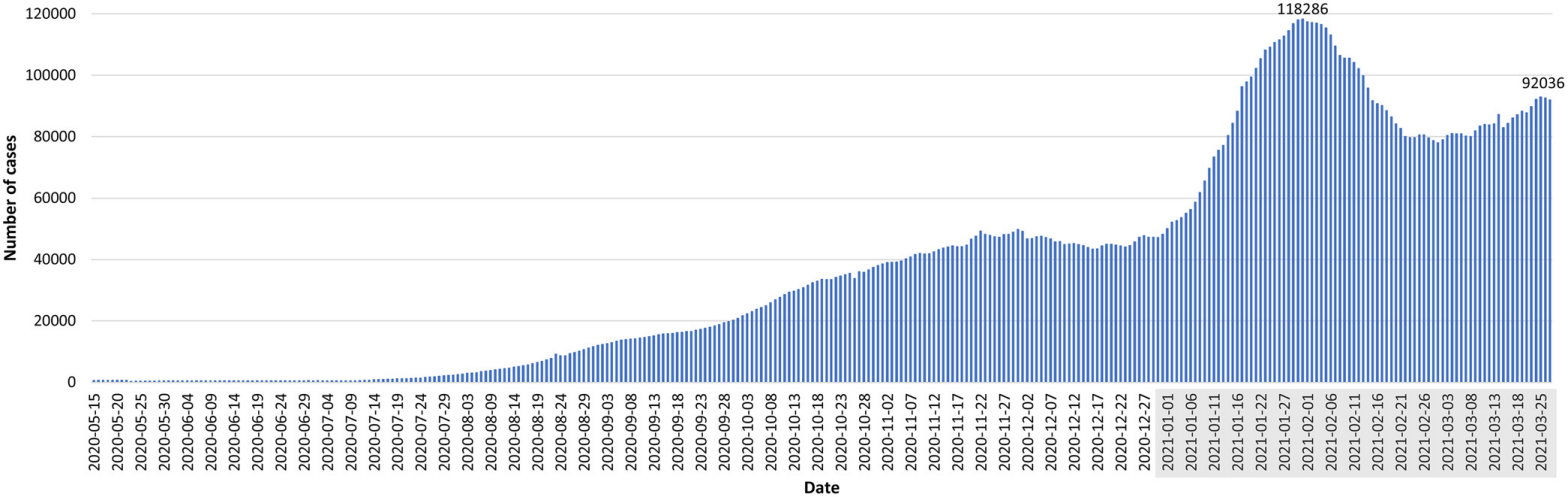
Distribution of active COVID-19 cases in Lebanon. Data shown covered the period between 15 May 2020 to 27 March 2021 (11) (https://www.emro.who.int/countries/lbn/index.html and https://twitter.com/WHOLebanon). Only dates with reported active cases were included. 2021 dates are shown shaded in gray.

**FIG 5:**
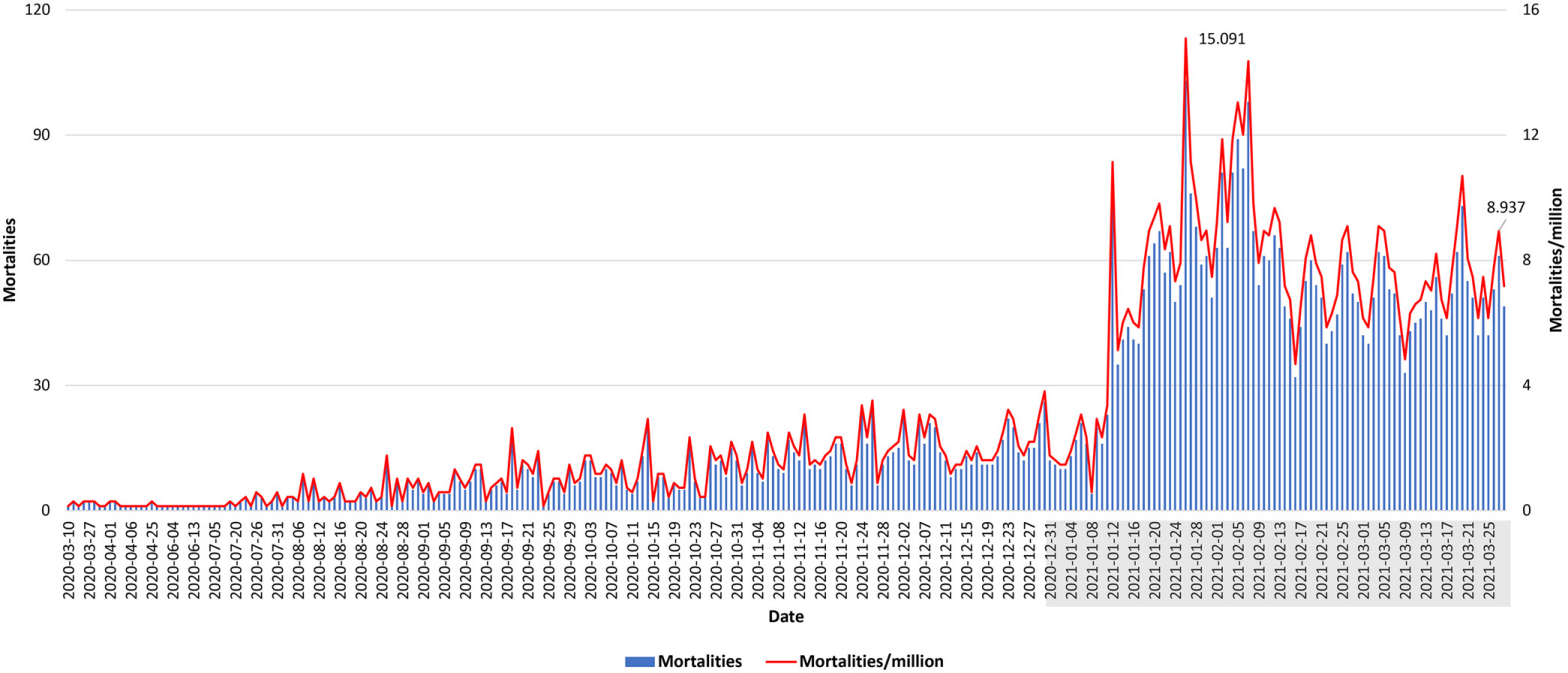
Number of daily COVID-19-related mortalities and mortalities per million over the year. Data shown covered the period between 10 March 2020 to 28 March 2021 (11) (https://www.emro.who.int/countries/lbn/index.html and https://twitter.com/WHOLebanon). Only dates with reported mortalities were included. 2021 dates are shown shaded in gray.

In December 2020, during the holiday season, measures were eased again. Restaurants were reopened, and all lockdown restrictions were removed with a concomitant increase in the daily number of reported COVID-19 cases reaching 1,843/day on 31 December 2020. The first report of the SARS-CoV-2 B.1.1.7 United Kingdom (UK) variant in Lebanon coincided with a surge in the number of cases. This was in line with the subsequent rise in positivity rates leading for the first time to a large-scale community transmission. January 2021 witnessed the highest daily morbidities and mortalities since the beginning of the pandemic. The reported positivity rate reached 32%, with a nationwide 90% occupancy in the intensive care unit (ICU) beds (Fig. 3, 4, and 5), calling for a strict and full lockdown as of 15 January 2021. One month into the January lockdown, the average positivity rate remained above 20%. Despite an alarming rise in the number of reported cases, a gradual lift and easing of the lockdown measures in February were implemented. This coincided with the launching of the first vaccination campaign.

## INSIGHTS

With an increase in the daily cases, COVID-19-related deaths, and ICU bed occupancy, the pandemic is far from being on a tight rein. The effectiveness of the COVID-19 mitigation strategies could not be separated from the ongoing economic, political, and health sector collapse. Increasing waves of positive cases followed the Beirut Port explosion, the reopening of the Beirut airport, and easing after each lockdown, leading over time to higher records in the total number of COVID-19-positive cases (9).

Genomic surveillance of SARS-CoV-2 is critical in monitoring and tracking the circulating viral lineages (10). Implementing an effective and rapid genomic surveillance of SARS-CoV-2 is challenging due to the scarcity of functional sequencing facilities, lack of support and funding, high costs and delivery delays, and lack of skilled personnel.

Looking through the first year of the pandemic in Lebanon helped in uncovering the gaps and lessons learned. Testing and molecular surveillance should be part of any plan to lift or impose confinement. Additionally, scaling up vaccination, preparedness, and response capacities in Lebanon is essential to mitigate viral spread, with the WHO, CNRS-L, NGOs, and humanitarian responses being a safety net to provide the much-needed support during the current pandemic. Finally, there is an urgent need to plan, strategize, and strengthen the health care system.
